# Sensory Stimulation of Oxytocin Release Is Associated With Stress Management and Maternal Care

**DOI:** 10.3389/fpsyg.2020.588068

**Published:** 2021-01-18

**Authors:** Toku Takahashi

**Affiliations:** ^1^Department of Surgery, Medical College of Wisconsin, Milwaukee, WI, United States; ^2^Integrative Medicine, Clinic Toku, Nagoya, Japan

**Keywords:** acupuncture, caesarian section (CS), epidural anesthesia, gastrointestinal (GI) motility, uterine contraction, HPA axis

## Abstract

It has been shown that various types of stress initiate different physiological and neuroendocrine disorders. Oxytocin (OT) is mainly produced in the supraoptic nucleus (SON) and paraventricular nucleus (PVN) of the hypothalamus. Hypothalamic OT has antistress effects and attenuates the hypothalamic–pituitary–adrenal (HPA) axis. One mechanism behind the antistress effects of OT is mediated through the inhibition from GABA_A_ receptors on corticotropin-releasing factor (CRF) expression at the PVN. Various manual therapies such as acupuncture, transcutaneous electrical nerve stimulation (TENS), and massage initiate the stimulation of somatosensory neurons of the body. It is well-known that TENS simulates OT expression, while it inhibits CRF expression at the PVN following chronic stress loading in rodents. Upregulation of OT expression at the hypothalamus is activated by the somatosensory stimulation, which is mediated via the spinothalamic pathway (the connection between the spinal cord and hypothalamus). Thus, somatosensory stimulation is beneficial in treating stress-associated symptoms. Hypothalamic OT is associated with the social behaviors, including maternal care and affiliation. Childhood neglect and/or child abuse are severely responsible for deleterious long-term effects on the cognitive/social activity and behavioral development. At parturition, a profound amount of OT is released into the systemic circulation in response to vaginal and cervical stimulation caused by the body of fetus, which induces the onset of maternal behavior. Peridural anesthesia effectively impairs the sensitivity to vaginal and cervical stimulation at parturition. OT levels in cerebrospinal fluid is significantly reduced following peridural anesthesia. The vaginal delivery mothers had significantly more OT pulses than the caesarian section (CS) mothers. Due to low levels of endogenous OT, maternal behavior could be interrupted by epidural anesthesia and CS at parturition because of the reduction of the usual sensory input from the genitalia.

## Importance of Hypothalamic Oxytocin in Stress

Oxytocin (OT) is mainly produced in the supraoptic nucleus (SON) and paraventricular nucleus (PVN) of the hypothalamus. In addition to the female reproductive function, OT plays important roles in attenuating stress responses and anxiety (Takahashi et al., 2013). OT inactivates the hypothalamic–pituitary–adrenal (HPA) axis in response to various stress stimuli (Neumann, 2008).

**Figure 1:**
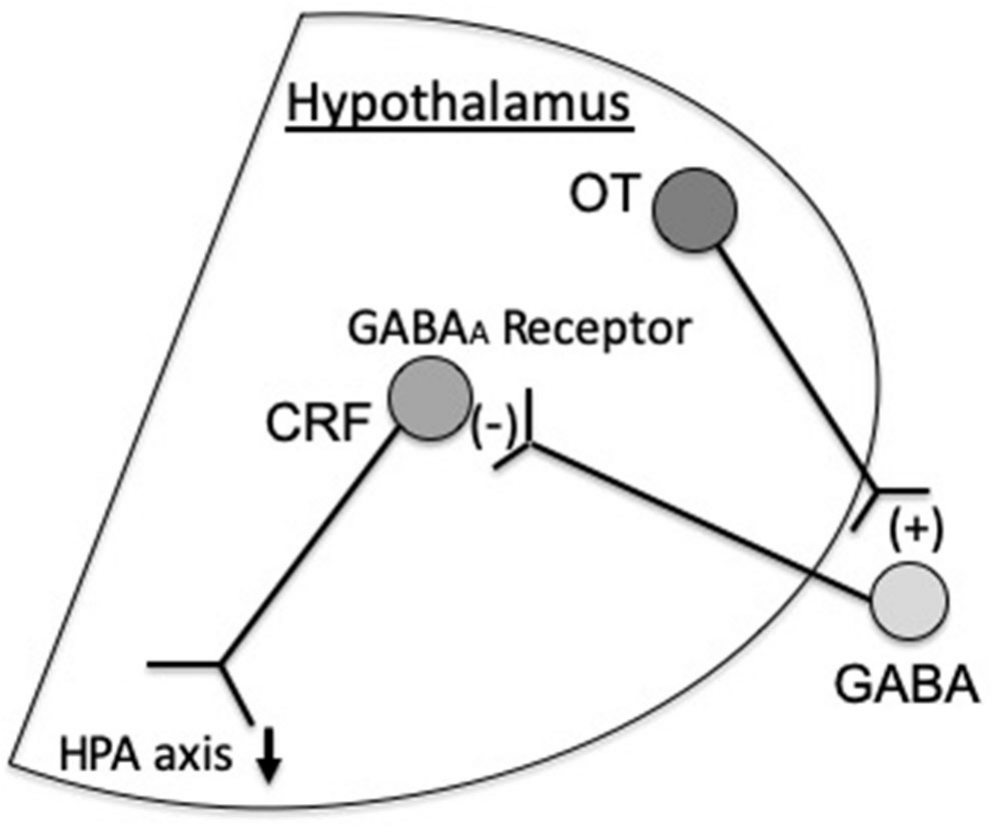
Oxytocin (OT) inhibits corticotropin-releasing factor (CRF) expression via GABA_A_ receptors at the paraventricular nucleus (PVN), which inactivates the hypothalamic–pituitary–adrenal (HPA) axis. In addition, others suggested that hypothalamic OT stimulates the production of alpha 2-adrenoreceptors in the nucleus tractus solitarius (NTS) and locus coeruleus (LC) on noradrenergic neurons projecting to the CRF neurons, which suppress CRF expression (Uvnäs-Moberg et al., 2015).

OT inhibits corticotropin-releasing factor (CRF) mRNA expression at the hypothalamus, resulting in antistress and anti-anxiety effects (Windle et al., 2004). It has been demonstrated that the inhibitory effect of OT on CRF mRNA expression is not a direct one on CRF neurons. GABAergic neurons are present in the surroundings of the PVN (peri-PVN). These GABA-projecting neurons into the PVN inhibits CRF expression via GABA_A_ receptors (Huber et al., 2005). Our previous study demonstrated that OT indirectly inhibits CRF mRNA expression via GABA_A_ receptors at the PVN (Bulbul et al., 2011) (Figure 1). Others also showed that OT activates GABAergic transmission in the central amygdala (Huber et al., 2005).

In addition, Uvnäs-Moberg et al. suggested that projection of hypothalamic OT enhances the activity of alpha 2-adrenoreceptors of the NTS (nucleus tractus solitarius) and LC (locus coeruleus), which then can influence the noradrenergic neurons projecting to the CRF neurons in the hypothalamus (Uvnäs-Moberg et al., 2015) (Figure 1). Thus, it seems reasonable that the inhibitory effect of OT on CRF expression is mediated via GABA_A_ receptors and alpha 2-adrenoreptors.

It has been demonstrated that restraint stress accelerates colonic transit, while it delays gastric emptying of the gastrointestinal (GI) tract in rodents. Accelerated colonic transit and delayed gastric emptying induced by stress are attenuated by intracerebroventricular (icv)-injection of OT in rodents (Zheng et al., 2010; Yoshimoto et al., 2012).

Central, but not peripheral, administration of OT markedly improved delayed gastric emptying induced by restraint stress in mice (Babygirija et al., 2010) and rats (Zheng et al., 2010). Similarly, aggravated colonic motility induced by water avoidance stress was attenuated by the central injection of OT (Matsunaga et al., 2009). During the recovery period following restraint stress loading, microdialysis study showed a significant increase in central OT levels in rats (Babygirija et al., 2012).

Thus, central OT plays an important role in regulating stress-induced GI function. Although OT and its receptors are shown to be present in the smooth muscle cells, enteric neurons, and mucosa in the GI tract (Welch et al., 2009), peripherally released OT from these cells may not have a major role in mediating antistress effects.

### Antistress Effect of Somatosensory Stimulation

**Figure 2:**
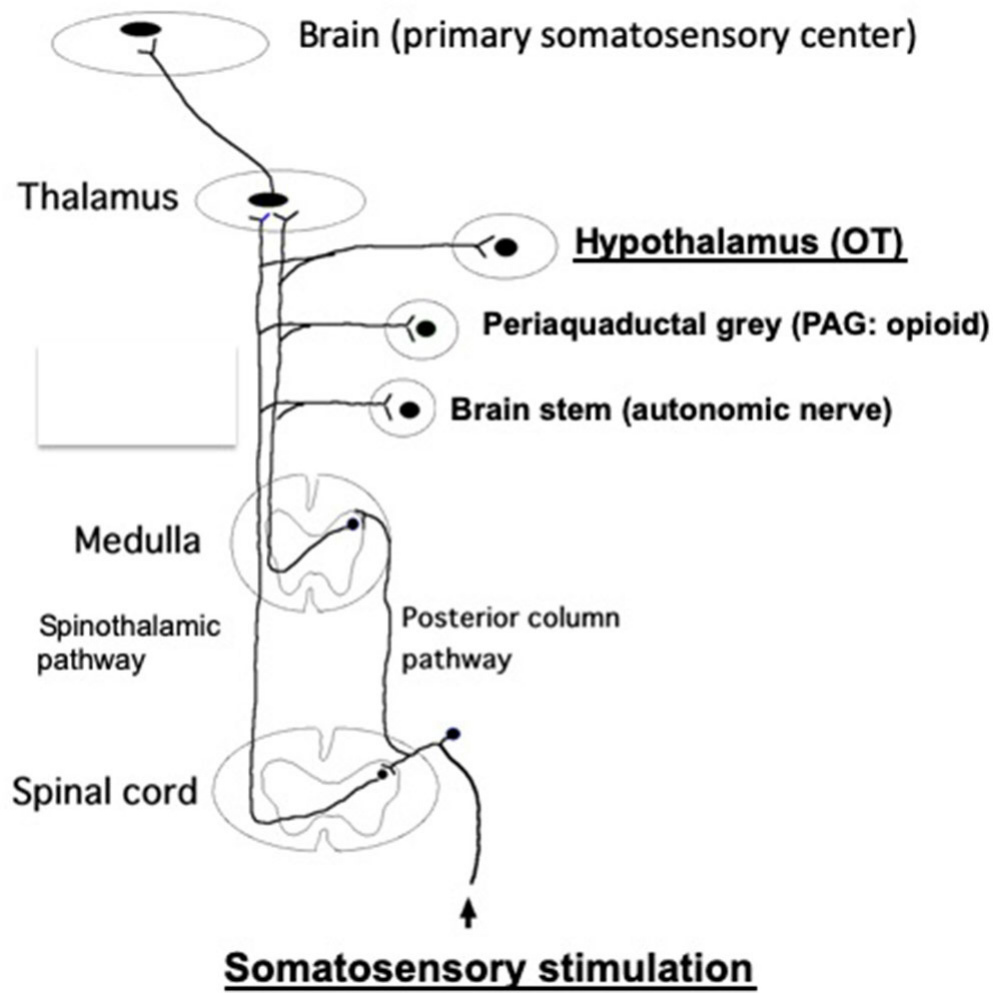
The posterior column pathway and spinothalamic pathway, which are responsible for the spinal–supraspinal pathways following somatosensory stimulation. These impulses are sent to the primary somatosensory cortex, through the thalamus. In addition, these impulses are relayed to other brain areas (brain stem, PAG, hypothalamus, etc.) via the collateral connections. Somatosensory stimulations, such as TENS, acupuncture, massage and skin touch, etc., promote antistress effects via stimulating somatosensory pathway, finally activating OT system at the hypothalamus. It has been shown that both somatic myelinated/unmyelinated afferent fibers mediate sympathetic nerve activities (Isa et al., 1985). Further studies are needed whether afferent myelinated and/or unmyelinated fibers are involved to mediate OT release from the brain. It remains unknown which afferent fibers are involved in the posterior column pathway and spinothalamic pathway.

It has been demonstrated that somatic afferent neurons originating from the skin and muscle are associated with the control of various autonomic nerve functions in rats and humans (Takahashi, 2013). The spinal–supraspinal pathways are responsible for somatosensory stimulation, which are mainly comprised of the posterior column pathway and spinothalamic pathway. The discriminative touch and sense of vibration activates peripheral thick myelinated afferent fibers, which then enter the ipsilateral posterior column pathway (dorsal column-medial lemniscus tract) and emerge into the contralateral spinothalamic pathway (Figure 2).

In contrast, the thinly myelinated or unmyelinated afferent fibers activated by mild temperature and pain are carried by the contralateral spinothalamic pathway. These impulses are further sent to the thalamus and ultimately sent to the primary somatosensory cortex. In addition, these impulses are also sent to other brain areas, including the hypothalamus, periaqueductal gray (PAG), brain stem, etc., via collateral connections (Hendelman et al., 2010) (Figure 2).

Somatosensory neurons are stimulated by the various manual therapies, including acupuncture, transcutaneous electrical nerve stimulation (TENS), and massage. Acupuncture utilizes the procedure of insertion of sharp thin needles into the skin and underlying muscle layer. Inserted needles are sometimes stimulated by electricity under various frequencies [1–100 Hz; electroacupuncture (EA)]. In contrast to acupuncture, TENS does not involve the procedure of needle insertion; instead, electrodes are placed on the skin and stimulated by electricity with various frequencies (1–100 Hz).

Acupuncture and/or TENS have been applied for treating various GI diseases, including constipation, diarrhea, functional dyspepsia (FD), and irritable bowel syndrome (IBS) (Iwa et al., 2006; Takahashi, 2011). Previous studies showed that TENS applied to the acupuncture points at the hands and lower legs improved gastric symptoms in patients with FD (Liu et al., 2008).

Animal studies demonstrated that TENS and EA improved various stress-associated motor responses of the GI tract. Accelerated colonic transit and delayed gastric emptying induced by restraint stress are improved by EA at the lower legs in rodents (Iwa et al., 2006). It is well-known that autonomic neuronal function is disorganized by acute and/or chronic stress loading. Our previous study showed that EA augmented the parasympathetic activity and inhibited the sympathetic activity under the acute stress loading in rats (Imai et al., 2009). Others showed that EA attenuated the incidents of stress-induced defecation and reduced CRF expression at the hypothalamus in the rat IBS model (Ma et al., 2009).

### Antistress Effect of Somatosensory Stimulation Is Mediated via Oxytocin

As pleasant touch increases the release of OT (Uvnas-Moberg et al., 1993), the pathophysiological effects of OT has been extensively studied with regard to its effects on mental activities and prosocial behavior (Koppel et al., 2017).

It has been suggested that OT levels in plasma as well as cerebrospinal fluid are increased by the various types of somatosensory stimulation (touch, massage, EA, thermal stimulation, and vibration) in anesthetized rats (Uvnas-Moberg et al., 1993). Others showed that massage-like stroking on the abdomen reduced blood pressure in anesthetized rats. As exogenously applied oxytocin diminished the effect of stroking, oxytocin system may exert an inhibitory effect on the blood pressure reflex (Kurosawa et al., 1995). These raise the possibility that TENS/EA/acupuncture may activate OT neurons at the hypothalamus, via somatosensory stimulation.

We have demonstrated that accelerated colonic transit induced by restraint stress is significantly attenuated by TENS in rats, which was abolished by the pretreatment with OT antagonists (icv-injection). In addition, we showed that TENS increased the number of OT-immunopositive neurons, while it decreased the number of CRF-immunopositive neurons at the hypothalamus following restraint stress in rats (Yoshimoto et al., 2012).

These strongly suggest that hypothalamic OT neurons are activated by the somatosensory stimulation, which is mediated via the spinothalamic pathway (Figure 2). Activated OT neurons, in turn, reduce CRF expression. This may promote the improvement of stress responses of GI tract.

It has been demonstrated that OT has special effects of decreasing background anxiety. Once OT neurotransmission is increased during the traumatic event, the formation of aversive memories will be prevented. Animal studies showed that when OT is administered prior to the training of fear conditioning or extinction, fear expression (the responses and behaviors to fear) was decreased in rats (Missig et al., 2010).

In human cases, the management of anxiety/stress and social functioning are often impaired in patients with posttraumatic stress disorder (PTSD).

Clinical trials showed that acupuncture is effective to treat the symptoms of patients with PTSD. People diagnosed with PTSD were divided into two groups: either an acupuncture treatment group or a cognitive–behavioral therapy (CBD) group. The study demonstrated that the acupuncture group provided a significant effect to improve their symptoms, compared with CBT group in PTSD patients (Hollifield et al., 2007).

Although there is no direct clue how acupuncture is effective for PTSD patients, our animal studies suggest that OT is likely to be released in response to acupuncture in these patients.

Traumatic stressful events activate the sympatho-adrenal system and the HPA axis. As mentioned before, OT can inhibit these systems, resulting, for example, in a reduction of cortisol levels. Thus, oxytocin may reduce fear responses and increase social functioning in patients exposed to severe stress, for example, in PTSD.

Although there are studies showing that oxytocin spray may decrease anxiety in humans, the recent systematic review of the randomized controlled trials (RCTs) demonstrated that the effect of OT spray on anxiety and depressive symptoms is still inconclusive (De Cagna et al., 2019).

We propose that TENS and/or acupuncture promote antistress effects via stimulating somatosensory pathway, finally activating OT neurons at the hypothalamus (Figure 2). As upregulated OT is responsible for antistress effects, somatosensory stimulation may have an important beneficial role in treating stress-associated various symptoms and diseases (Takahashi, 2013).

## Importance of Sensory Stimulation of Oxytocin Release During Parturition

In placental mammals, the newborn infants are totally dependent upon nutrition (their mothers' milk). In addition, the sustained close relationship with their mothers is essential to maintain the body temperature for survival.

The bonding between the mother and infant is important in its influence on the infant's future. Child neglect and/or abuse during early life of the infant may develop abnormal mother–infant bonding systems, which may cause inappropriate social functions and/or behavioral activity later in life (Hildyard and Wolfe, 2002).

During pregnancy, OT expression is increased not only in the SON and the PVN, but also in other nuclei (Caldwell et al., 1987; Ludwig, 1998). At parturition, a profound amount of OT is released into the systemic circulation in response to vaginal and cervical stimulation caused by the fetus. During vaginal delivery, OT is substantially released into the systemic circulation from the posterior pituitary. Peripherally released OT induces uterine contractions during parturition through the activation of OT receptors of uterine smooth muscle cells.

OT is also released centrally to the surrounding brain nuclei (Gimpl and Fahrenholz, 2001). Once rats are received, the lesions at the PVN area during pregnancy, maternal behavior is significantly impaired in post-parturient (Insel and Harbaugh, 1989). In female rats that received an OT antagonist immediately after parturition, maternal behavior is significantly disturbed (van Leengoed et al., 1987). Thus, the hypothalamic OT-neuronal system plays an important role to initiate maternal behavior following delivery.

The skin-to-skin contact alter birth initiates maternal, vocal, and tactile interaction between the mother and infant. Because the mother becomes calmer and plasma cortisol levels drops, it is suggested that the antistress effects are induced by skin-to-skin contact. Oxytocin release in the brain is likely to lie behind these behavioral and physiological effects (Uvnäs-Moberg et al., 2015).

### Peridural Anesthesia

It has been suggested that released OT in response to vaginal and cervical stimulation during parturition promotes nurturing behaviors and facilitates the mother–infant bonding after the delivery.

Peridural anesthesia effectively impairs the sensitivity to vaginal and cervical stimulation at parturition. In order to study the effects of peridural anesthesia on maternal behavior, sheep studies were performed. Pregnant sheep received either the early peridural anesthesia procedure (EP) or the late peridural anesthesia procedure (LP). When peridural anesthesia was performed at the late stage of parturition (LP), maternal behavior was only slightly altered, compared with controls (without peridural anesthesia). In contrast, maternal behavior was severely impaired in the case of EP.

Seven out of eight mothers failed to show any interest to their newborn babies in 30 min after the delivery, in the case of EP (Krehbiel et al., 1987). These suggest that the genital stimulation is an important factor for the rapid onset of maternal behavior in sheep.

The concentration of OT in cerebrospinal fluid is significantly reduced following peridural anesthesia, suggesting inhibition of the central release of OT during parturition (Krehbiel et al., 1987). It is highly likely that vaginal and cervical stimulation caused by the fetus activates OT system, which initiates the onset of maternal behavior.

Because stimulation of the uterine afferent nerves excites the neuronal activity at the PVN, specific sensory afferents may reach the PVN from the uterus. In addition, somatic afferents converge in the hypothalamus (Akaishi et al., 1988).

Electrophysiological study showed that pelvic nerves and hypogastric nerves are activated by the passage of the fetus down the vagina and cervices (birth canal) (Peters et al., 1987). Stimulation of the uterine afferent nerves, the pelvic nerves, and hypogastric nerves, increases the activity of the presumed OT neurons at the PVN (Akaishi et al., 1988).

Maternal behavior could be interrupted by epidural anesthesia at the parturition due to low levels of endogenous OT because of the reduction of usual sensory input from the genitalia. Thus, normal vaginal delivery may be important to develop maternal behavior in every mammal, including humans. One reason for the hypersecretion of OT during vaginal delivery, besides causing uterine contraction, is to prepare a potent mother–infant bonding (Uvnäs-Moberg et al., 2015).

### Cesarean Sections

Released OT from the posterior pituitary induces uterine contractions during parturition, which helps the vaginal delivery. As OT plays an important role in the maternal care of newborn babies, the likely reason for the hypersecretion of OT during vaginal delivery is to prepare for the onset of maternal behavior for newborns. In other words, active labor and delivery stimulate the rapid release of endogenous OT, which helps in mother–infant bonding.

The age when women began to opt for elective cesarean sections (CSs) on request was a turning point in the history of childbirth. Today, in many countries, most women opt to give birth by elective CS on request, which may cause a reduction in releasing endogenous OT.

Is there any difference of hormonal pattern of OT between the women delivered by the vaginal route and the women delivered by emergency CS? Does the different pattern of OT release show any relation to the duration of breastfeeding?

To answer these questions, Nissen et al. (1996) compared the plasma level of OT and prolactin in 17 mothers with emergency CS and 20 mothers with normal vaginal delivery, in connection with breastfeeding on day 2 postpartum.

The plasma level of OT was significantly higher in the vaginal delivery mothers than those of the CS mothers. Furthermore, the CS mothers showed no significant rise in prolactin levels at 20–30 min after the onset of breastfeeding (Nissen et al., 1996).

However, it remains unknown whether CS women with reduced OT release have problems associated with mother–infant bonding. Further clinical studies are needed to investigate whether CS impairs the development of maternal behavior in humans (Takahashi et al., 2013). Further studies are also needed whether emergency CS may have a different OT pattern than planned CS.

## Author Contributions

The author confirms being the sole contributor of this work and has approved it for publication.

## Conflict of Interest

The author declares that the research was conducted in the absence of any commercial or financial relationships that could be construed as a potential conflict of interest.
